# One-Pot Ugi/Aza-Michael Synthesis of Highly Substituted 2,5-Diketopiperazines with Anti-Proliferative Properties

**DOI:** 10.3390/molecules171214685

**Published:** 2012-12-11

**Authors:** Andreas Hartung, Florian Seufert, Carsten Berges, Viktoria H. Gessner, Ulrike Holzgrabe

**Affiliations:** 1 Institute of Pharmacy and Food Chemistry, University of Wuerzburg, Am Hubland, 97074 Wuerzburg, Germany; E-Mails: andreas.hartung@uni-wuerzburg.de (A.H.); f.seufert@pharmazie.uni-wuerzburg.de (F.S.); 2 Department of Internal Medicine II, Translational Immunotherapy, University Hospital Wuerzburg, Versbacherstr 5, 97078 Wuerzburg, Germany; E-Mail: berges_c@medizin.uni-wuerzburg.de; 3 Institute of Inorganic Chemistry, University of Wuerzburg, Am Hubland, 97074 Wuerzburg, Germany; E-Mail: vgessner@uni-wuerzburg.de

**Keywords:** multicomponent Ugi-type reaction, intramolecular Michael addition, 2,5-diketopiperazines, anti-proliferative effects

## Abstract

The well-known Ugi reaction of aldehydes with amines, carboxylic acids and isocyanides leads to the formation of acyclic α-acylaminocarboxamides. Replacement of the carboxylic acid derivatives with β-acyl substituted acrylic acids gives access to highly substituted 2,5-diketopiperazines in one single reaction-step without additives or complex reaction procedures. The obtained diketopiperazines show anti-proliferative effects on activated T cells and represent therefore potential candidates for targeting unwanted T cell-mediated immune responses.

## 1. Introduction

Nowadays, multicomponent reactions (MCRs) are part of the conventional repertoire of synthetic chemists for a couple of good reasons. In contrast to a sequential multi-step approach, MCRs usually give more or less complex product molecules out of three or more starting materials in one single step. In consequence the reaction times and the unavoidable efforts on the reaction and the workup procedure are significantly reduced. However, the most important advantage of multicomponent reactions in academic and industrial research is their variability [1]. By consecutively changing the structures of the starting compounds, a diverse library of numerous compounds can be created very quickly. As this feature correlates perfectly with the demands of medicinal chemistry, MCRs are notably utilized in lead structure optimization. E.g., starting from xylocaine [2], which is an important local anaesthetic and accessible within just one step, numerous analogue compounds have been synthesized in the same way and are now commercially available anaesthetic drugs [1,3].

**Figure 1:**
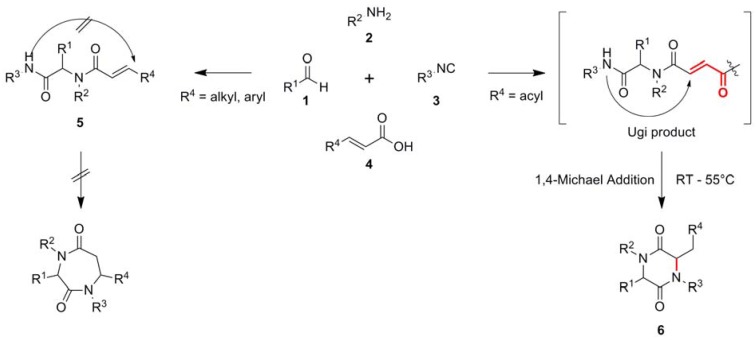
The U-4CR reaction with α,β-unsaturated carboxylic acids gives the expected Ugi product **5**, whereas the usage of β-acyl substituted acrylic acid gives rise to highly substituted 2,5-diketopiperazines **6**.

In an effort to synthesize a smart library of Ugi products with potential antitumoral activity (unpublished results) we started off with the well-known Ugi four-component (U-4CR) reaction using acrylic acids, to obtain highly diverse Michael systems **5** [4]. However, introducing β-acyl substituted acrylic acid derivatives influences the reaction outcome and gives access to the pharmacological attractive scaffold of 2,5-piperazindiones **6** (2,5-DKPs) [5,6,7], without any heating or microwave irradiation (see Figure 1). Preliminary pharmacological investigations suggest the synthesized DKPs are promising candidates for drugs with anti-proliferative activity.

## 2. Results and Discussion

While highly substituted 2,5-DKPs are conventionally synthesized via classic dipeptide formation [8,9] or dipeptide synthesis via the Ugi reaction [7,10], both followed by a deprotection and cyclization step, a direct one-pot synthesis pathway has recently attracted attention [11]. An aldehyde [12] or keto compound [13] can be converted with an amine, isocyanide and β-acyl substituted acrylic acid under microwave irradiation necessary to promote an Aza-Michael ring closure. It was reported that this reaction can be applied to various alkyl- and benzyl isocyanides which are starting materials known to give reasonable yields in the Ugi reaction.

Although the use of *p*-toluenesulfonylmethyl isocyanide (TosMIc) is more discerning due to its low reactivity [14,15], we decided to extent the scope of the reaction in this way for two reasons: (1) highly lipophilic compounds tend to address many targets unspecifically and increase promiscuity [16] and (2) compounds need to be water soluble for *in vitro* and *in vivo* studies. TosMIc therefore is highly appropriate as it provides a polar and metabolic fairly inert sulfone function. Here we report the synthesis of a couple of structurally different tosyl substituted 2,5-DKPs with anti-proliferative properties according to the elegant multicomponent pathway by thermal means or even without any heating.

The reaction of 2-bromobenzyaldehyde (**1a**), 3-(aminomethyl)pyridine (**2a**), TosMIc (**3**) and cinnamic acid (**4a**) gave the desired acyclic Ugi-product **5** as expected (Figure 1). However, replacing the carboxylic acid component by 3-benzoylacrylic acid (**4b**–**d**)–which just differs in one carbonyl group–resulted in the ring closed 2,5-diketopiperazine product **6a** (Figure 1, Table 1). The key step is an intra-molecular aza-1,4-Michael addition, classified as a 6-exo-trig reaction. In contrast, compound **5** and several analogues (not shown), also containing a Michael-system, do not undergo the 7-endo-trig reaction to form the corresponding 7-membered bislactam ring under comparable conditions (Figure 1). Hence, the essential structural moiety for the cyclisation reaction is the double Michael system of the butenedione, which can be introduced by any β-acyl substituted acrylic acid derivative.

In order to investigate the initially observed anti-proliferative properties in more detail, a small series of different derivatives was synthesised (Table 1). It is noteworthy that all reactions were carried out between room temperature and 55 °C by thermal heating. In contrast to a recently published work mainly using fumaric acid in the synthesis of 2,5-DKPs [12], no microwave irradiation was necessary for the considered reactions. Moreover, the syntheses of the products reported here could not take advantage of being carried out under the influence of microwaves or of using water as solvent. The moderate yields obtained are in similar range as reported for related multicomponent reactions employing TosMIc [17].

**Figure 2:**
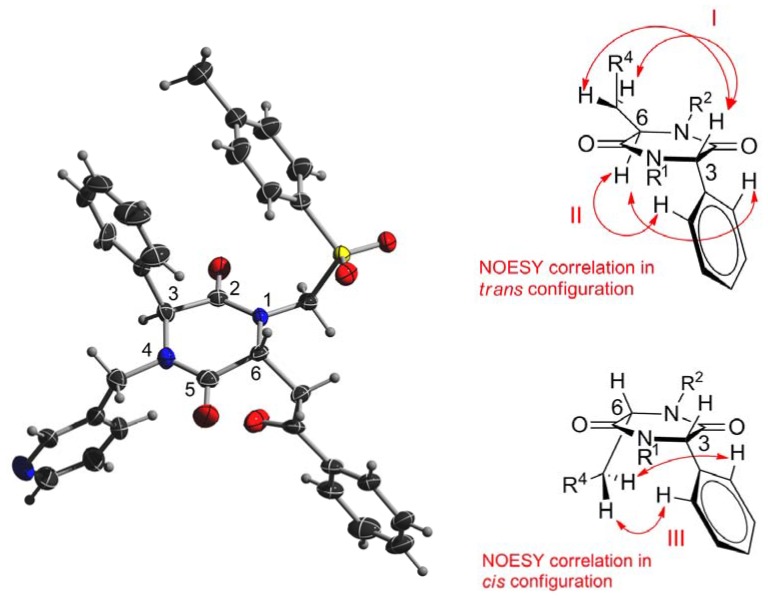
The X-ray crystal structure of **6b** shows the *trans* substitution at C-3 and C-6 of the DKP (left). NOESY correlations indicate the relative configuration at the DKP core. I, II: *trans*; III: *cis* (right).

The X-ray crystal structure of **6b** (Figure 2) not only confirmed the structure deduced from NMR spectroscopy, but also provided information about the relative configuration. In accordance to the NMR analyses, the substituents at C-3 and C-6 of the almost flat DKP ring are in a *trans*-configuration. When *trans*-oriented, the dipolar coupling mediated proton-proton correlation–known as Nuclear Overhauser Effect (NOE)–was observed between the proton at C-3 and the methylene protons attached to C-6 (Figure 2, correlation I) and/or between the proton at C-6 and the *ortho*-protons of the phenyl substituent at C-3 (Figure 2, correlation II). In contrast, when the substituents are in the *cis*-position, a NOE correlation was observed between the methylene protons at the substituent of C-6 and the *ortho*-protons of the phenyl ring at C-3 (Figure 2, correlation III) [18]. While the majority of the synthesized compounds precipitated readily out of the reaction mixtures, compounds **6a**, **6c** and **6d** needed purification by means of column chromatography. Interestingly, by precipitation only one of the two possible diastereomers was isolated. Moreover, the filtrate of two representatively investigated reaction mixtures (compounds **6b** and **6j**) did not contain any *cis*-isomer. The synthesis of compounds **6a**, **6c** and **6d** gave *cis*:*trans* diastereomeric ratios ranging from 1:1 to 1:2 (determined using the integrals of the NMR signals of the hydrogens attached to C-3) and were separated by means of double column chromatography.

2,5-DKPs have been shown to exhibit anticancer activity by inhibiting proliferation of cancer cell lines [19,20]. In order to analyze the anti-proliferative effect of 2,5-DKPs we used activated T cells because T cells are not only key cells for the initiation of an adaptive immune response, but also participate in the onset of dysregulated immune responses like inflammatory diseases, autoimmune diseases and transplant rejection [21,22]. Thus we aimed to clarify if 2,5-DKPs can also be used for treatment of these unwanted T cell-mediated immune responses. For comparative purposes, heat shock protein 90 inhibitor 17-dimethylaminoethylamino-17-demethoxygeldanamycin (17-DMAG) which selectively blocks proliferation of activated T cells, was included in the assay [23].

**Figure 3:**
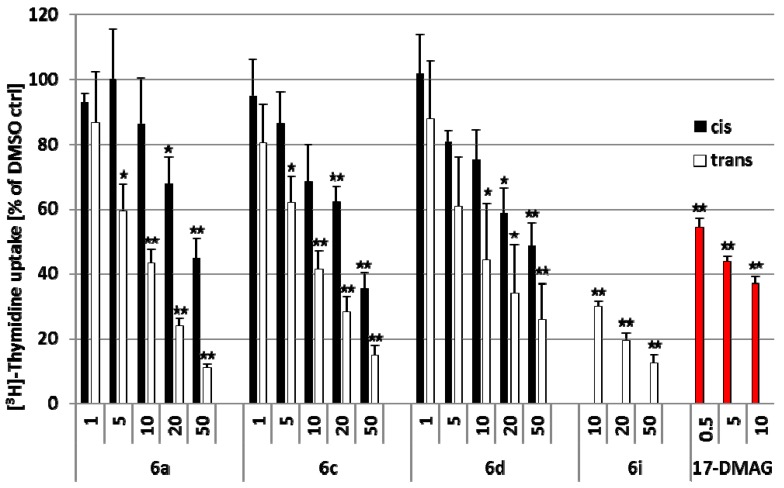
Purified human T cells were activated by coculture with allogeneic human DCs for 5 days. Activated human T cells were exposed for the last 24 h of coculture to the indicated concentrations [µM] of synthesized *trans* compounds (white bars), *cis* compounds (black bars), 17-DMAG (red bars) or DMSO ctrl, and incorporation of [^3^H]thymidine was determined as described in the Experimental section. Data are given as mean values ± standard error of the mean of four independent experiments carried out in triplicate. * *p* < 0.05, ** *p* < 0.01 compared to DMSO control.

T cells activated in a physiological manner by allogeneic dendritic cells (DC) were incubated for 24 h with the synthesized compounds, 17-DMAG and DMSO control and subsequently proliferation of activated T cells was determined by [^3^H]-thymidine uptake. As shown in Figure 3, the *trans* compounds **6a**, **6c**, **6d** and **6i** reduced significantly proliferation of activated T cells in a dose-dependent manner, confirming results of other studies which showed that 2,5-DKPs are able to suppress proliferation of highly proliferating cells of different origin [19,20].

High molecular concentrations (50 µM) of the 2,5-*trans*-DKPs **6a**, **6c**, **6d** and **6i** suppressed proliferation of activated T cells more intensively than the highest used 17-DMAG concentration (10 µM). However, this molecular concentration of 17-DMAG were more effective in inhibiting proliferation of activated T cells than equal concentrations of the *trans* compounds **6a**, **6c** and **6d**. In contrast, compounds **6b**, **6e**, **6f**, **6g** and **6h** and the *cis* compounds **6a**, **6c** and **6d** showed a lower or no anti-proliferative effect on activated T cells (Supplementary Table 1).

Analysing the structure-activity relationships of the synthesized compounds, bromo and chloro substitution in R^1^ proved to be favourable (Br ≈ Cl > I > H > F). With regard to the influence of R^2^, the polar pyridin-3ylmethyl and MeO-benzyl substituents are privileged over the lipophilic benzyl and phenethyl groups. Variations in R^4^ demonstrate a preference for the chlorobenzoyl residue (Cl-benzoyl > benzoyl > MeO-benzoyl).

To investigate the cytotoxicity we incubated resting T cells for 24 h with increasing concentrations of compounds **6a**–**j**, 17-DMAG and DMSO control and afterwards measured apoptosis induction by defining percentage of Annexin V+/7-AAD+ cells. We noticed that incubation of resting T cells with all used concentrations of compound **6d** (*trans*), **6e** and **6f** caused no apoptosis, whereas high molecular concentrations of compound **6a** (*cis* and *trans*), **6c** (*cis* and *trans*) and **6d** (*cis*) induced negligible apoptosis which is comparable to apoptosis rate induced by DMAG. In contrast, all used concentrations of compound **6g** induced a moderate level and of compound **6i** even a considerable level of apoptosis in resting T cells (see Supplementary Table 1). Thus, the observed anti-proliferative effect on activated T cells of compound **6g** and **6i** can be most likely attributed to cytotoxic activities.

To summarize, *trans*-compounds **6a**, **6c** and **6d** significantly inhibited proliferation of activated T cells and showed at high molecular concentrations an even more profound anti-proliferative effect than highest used 17-DMAG concentration, combined with negligible cytotoxicity. Drugs of this pharmacological profile are urgently needed.

## 3. Experimental

### 3.1. General

Melting points were determined by means of a Stuart melting point apparatus SMP11 (Bibby Scientific, Stone, UK) and not corrected. IR spectra were obtained using a JASCO FT/IR-6100 spectrometer (JASCO, Gross-Umstadt, Germany). TLC was performed on silica gel 60 F_254_-coated glass sheets (Merck, Darmstadt, Germany). ^1^H (400.132 MHz) and ^13^C (100.613 MHz) NMR spectra were recorded on a Bruker AV 400 instrument (Bruker Biospin, Ettlingen, Germany). As internal standard the signals of the deuterated solvent was used (DMSO-*d_6_*: ^1^H 2.50 ppm, ^13^C 39.52 ppm; CDCl_3_: ^1^H 7.26 ppm, ^13^C 77.16 ppm). Abbreviations for data quoted are: s, singlet; d, doublet; t, triplet; m, multiplet; b, broad; dd, doublet of doublets; ddd, doublet of doublet of doublets; dt, doublet of triplets. Coupling constants (J) are given in Hz. LCMS data were obtained using a Agilent 1100 Series LC/MSD Trap (Agilent Technologies, Böblingen, Germany). HRMS data were obtained using a Bruker micrOTOF focus II instrument (Bruker Daltonik, Bremen, Germany). The elemental analyses (CHN) were recorded on a EURO EA 3000 Elemental Analyzer instrument (Eurovektor Instruments, Hekatech, Germany). Chemicals were purchased from Aldrich (Steinheim, Germany) and Thermo Fisher Scientific (Geel, Belgium) and used without purification.

### 3.2. Procedure for the Synthesis of the Acyclic Ugi-Product

*N-(1-(2-Bromophenyl)-2-oxo-2-((tosylmethyl)amino)ethyl)-N-(pyridine-3-ylmethyl)cinnamamide* (**5**). To a solution of 2-bromobenzaldehyde (3 mmol, 350 µL) in absolute methanol (5 mL) 3-(aminomethyl)pyridine (3 mmol, 310 µL) was added and stirred for 20 min at room temperature. Then *p*-toluenesulfonyl-methyl isocyanide (3.6 mmol, 700 mg) and cinnamic acid (3 mmol, 450 mg) were added. After 96 h the solvent was evaporated, the residue dissolved in CHCl_3_ and extracted with a saturated aqueous solution of NaHCO_3_. The organic layer was dried over Na_2_SO_4_ and evaporated. The crude product was purified by means of column chromatography (silica gel 60, toluene/ethyl acetate 1:3) to give the pure **5**. Colorless amorphous solid (310 mg, 17%): mp 184–186 °C; IR *ν* 2952, 1689, 1643, 1594, 1415, 1320, 1288, 1138, 816, 762, 704 cm^−1^; ^1^H-NMR (400 MHz, CHCl_3_) δ 8.31 (d, 3.4 Hz, 1H), 8.10 (s, 1H), 7.85 (d, 15.3 Hz, 1H), 7.71 (d, 8.0 Hz, 2H), 7.51 (br. s, 1H), 7.40 (br. s, 3H), 7.36–7.31 (m, 2H), 7.27 (d, 8.0 Hz, 2H), 7.36–7.01 (m, 4H), 7.22–7.14 (m, 1H), 7.11–7.00 (m, 1H), 6.69 (d, 15.3 Hz, 1H), 6.54 (s, 1H), 4.91 (dd, 13.7 Hz, 7.3 Hz, 1H), 4.80 (d, 18.0 Hz, 1H), 4.63–4.49 (m, 2H), 2.39 (s, 3H); ^13^C NMR (100 M Hz, CHCl_3_): δ 169.6, 167.8, 147.7, 147.1, 145.6, 145.2, 134.6, 134.5, 134.0, 133.2, 132.9, 132.7, 131.3, 130.8, 130.2, 130.0, 128.9, 128.6, 128.2, 128.1, 127.8, 123.2, 116.4, 61.2, 60.3, 46.8, 21.7; LCMS (ESI^+^): *m*/*z* 619.5 [M+H]^+^; Anal. calcd. for C_31_H_28_BrN_3_O_4_S: C 60.20, H 4.56, N 6.79, S 5.18; found: C 60.04, H 4.60, N 6.88, S 5.01.

### 3.3. General Procedure for the Synthesis of Piperazine-2,5-diones ***6a***–***j***

To a solution of benzaldehyde derivative (1 eq.) in absolute methanol the corresponding amine (1 eq.) was added and stirred for 30 min at room temperature. Then *p*-toluenesulfonylmethyl isocyanide (1 eq.) and the corresponding benzoylacryclic acid derivative (1 eq.) were added. The solution was stirred until the reaction, controlled by means of TLC, was finished. If necessary, the reaction mixture was heated.

*3-(2-Bromophenyl)-6-(2-oxo-2-phenylethyl)-4-(pyridin-3-ylmethyl)-1-(tosylmethyl)piperazine-2,5-dione* (**6a**). Quantities: 2-bromobenzaldehyde (470 µL, 4.0 mmol), 3-(aminomethyl)pyridine (410 µL, 4.0 mmol), *p*-toluenesulfonylmethyl isocyanide (940 mg, 4.8 mmol), 3-benzoylacrylic acid (710 mg, 4.0 mmol). The solvent was evaporated *in vacuo* and the residue was purified by column chromatography (1st silica gel 60, *n*-hexane/ethyl acetate 1:20) to give the purified mixture of *cis*- and *trans*-product in a yield of 35%. A second purification step by column chromatography (2nd flash silica gel, dichloromethane/methanol 10:0.5) gave the *cis*- and *trans*-diastereomers separately. ***trans*-6a**: Colorless amorphous solid (341 mg, 13%): mp 111–114 °C; IR *ν* 1657, 1596, 1422, 1319, 1140, 1085, 762, 688 cm^−1^; ^1^H-NMR (400 MHz, DMSO) δ 8.40 (dd, 4.8 Hz, 1.6 Hz, 1H), 8.06 (d, 1.8 Hz, 1H), 8.02 (dd, 7.8 Hz, 1.2 Hz, 2H), 7.71 (d, 8.1 Hz, 2H), 7.70 (tt, 7.8 Hz, 1.2 Hz, 1H), 7.58 (t, 7.8 Hz, 2H), 7.55 (dd, 7.7 Hz, 1.3 Hz, 1H), 7.37–7.29 (m, 2H), 7.35 (d, 8.1 Hz, 2H), 7.26 (td, 7.7 Hz, 1.7 Hz, 1H), 7.23 (dd, 7.8 Hz, 4.8 Hz, 1H), 7.13 (dd, 7.7 Hz, 1.7 Hz, 1H), 5.68 (s, 1H), 5.36 (d, 14.8 Hz, 1H), 4.97 (d, 14.8 Hz, 1H), 4.96–4.92 (m, 1H), 4.84 (d, 15.4 Hz, 1H), 4.33 (dd, 19.1 Hz, 1H), 3.92 (dd, 19.1 Hz, 1H), 3.74 (d, 15.4 Hz, 1H), 2.34 (s, 3H); ^13^C-NMR (100 MHz, DMSO): δ 197.6, 165.9, 164.5, 149.1, 148.4, 144.7, 135.8, 135.6, 135.2, 135.0, 133.7, 132.8, 130.9, 130.6, 130.3, 129.8, 128.6, 128.1 (2C), 128.0, 124.1, 123.1, 62.8, 62.4, 55.9, 45.0, 39.9, 21.0; HRMS (MS-TOF): [M+H]^+^ calcd. for C_32_H_29_BrN_3_O_5_S: 646.1006, found: 646.1001. ***cis*-6a**: Colorless amorphous solid (161 mg, 6%): mp 99–100 °C; IR *ν* 1657, 1595, 1422, 1323, 1140, 1084, 761, 688 cm^−1^; ^1^H-NMR (400 MHz, DMSO) δ 8.48 (dd, 4.7 Hz, 1.7 Hz, 1H), 8.24 (d, 1.7 Hz, 1H), 8.10 (d, 9.3 Hz, 1.2 Hz, 2H), 7.84 (dd, 7.8 Hz, 1.3 Hz, 1H), 7.70 (tt, 7.4 Hz, 1.2 Hz, 1H), 7.63–7.56 (m, 4H), 7.56 (dd, 7.8 Hz, 1.0 Hz, 1H), 7.51 (dt, 7.9 Hz, 1.7 Hz, 1H), 7.45 (td, 7.8 Hz, 1.0 Hz, 1H), 7.34 (dd, 7.9 Hz, 4.7 Hz, 1H), 7.27 (td, 7.8 Hz, 1.3 Hz, 1H), 7.19 (d, 8.1 Hz, 2H), 5.24 (s, 1H), 5.06–5.00 (m, 1H), 5.12 (d, 14.7 Hz, 1H), 5.01 (d, 14.7 Hz, 1H), 4.87 (d, 15.5 Hz, 1H), 4.21 (dd, 18.7 Hz, 4.4 Hz, 1H), 3.99 (dd, 18.7 Hz, 4.1 Hz, 1H), 3.76 (d, 15.5 Hz, 1H), 2.26 (s, 3H); ^13^C NMR (100 M Hz, DMSO): δ 196.9, 165.2, 163.9, 149.1, 148.6, 144.7, 136.1, 135.6, 135.0, 134.1, 133.4, 132.7, 131.1, 130.4, 129.8, 129.6, 128.6, 128.4, 128.3, 128.1, 124.7, 123.4, 63.9, 61.4, 56.0, 44.9, 40.6, 21.0; HRMS (MS-TOF): [M+H]^+^ calcd. for C_32_H_29_BrN_3_O_5_S: 646.1006, found: 646.1008.

*3-(2-Oxo-2-phenylethyl)-6-phenyl-1-(pyridin-3-ylmethyl)-4-(tosylmethyl)piperazine-2,5-dione* (***trans*-6b**). Quantities: benzaldehyde (900 µL, 8.9 mmol), 3-(aminomethyl)pyridine (910 µL, 8.9 mmol), *p*-toluenesulfonylmethyl isocyanide (1.74 g, 8.9 mmol), 3-benzoylacrylic acid (1.60 g, 8.9 mmol). The precipitate was filtered, dissolved in dichloromethane and crystallization initiated by adding diethyl ether to give pure ***trans*-6b**. Colorless solid (2.28 g, 45%): mp 204–205 °C; IR *ν* 2952, 1662, 1597, 1429, 1418, 1319, 1289, 1141, 1087, 757, 713, 691 cm^−1^; ^1^H-NMR (400 MHz, DMSO) δ 8.42 (dd, 4.9 Hz, 1.6 Hz, 1H), 8.17 (d, 1.6 Hz, 1H) 8.00 (d, 7.5 Hz, 2H), 7.69 (t, 7.5 Hz, 1H), 7.63 (d, 8.3 Hz, 2H), 7.57 (t, 7.5 Hz, 2H), 7.41 (dt, 7.5 Hz, 1.6 Hz, 1H), 7.39–7.30 (m, 1H), 7.33 (d, 7.8 Hz, 2H), 7.27 (d, 8.3 Hz, 2H), 7.25 (dd, 7.5 Hz, 4.9 Hz, 1H ), 7.08 (dd, 7.8 Hz, 1.2 Hz, 2H), 5.48 (d, 14.9 Hz, 1H), 5.20 (s, 1H), 4.93 (dd, 4.4 Hz, 3.5 Hz, 1H), 4.88 (d, 14.9 Hz, 1H), 4.86 (d, 15.3 Hz, 1H), 4.23 (dd, 18.9 Hz, 4.4 Hz, 1H), 3.88 (dd, 18.9 Hz, 3.5 Hz, 1H), 3.79 (d, 15.3 Hz, 1H), 2.34 (s, 3H); ^13^C-NMR (100 MHz, DMSO): δ 197.3, 165.7, 165.5, 149.0, 148.3, 144.7, 135.9, 135.7, 135.4, 134.6, 133.6, 131.4, 129.8, 128.6, 128.5, 128.3, 128.03, 127.97, 127.90, 123.1, 63.4, 61.7, 55.4, 45.2, 37.9, 21.0; LCMS (ESI^+^): *m*/*z* 567.4 [M]^+^; Anal. calcd. for C_32_H_29_N_3_O_5_S: C 67.71, H 5.15, N 7.40, S 5.65; found: C 67.39, H 5.11, N 7.33, S 5.54.

*3-(2-Chlorophenyl)-6-(2-oxo-2-phenylethyl)-4-(pyridin-3-ylmethyl)-1-(tosylmethyl)piperazine-2,5-dione* (**6c**). Quantities: 2-chlorobenzaldehyde (230 µL, 2.0 mmol), 3-(aminomethyl)pyridine (200 µL, 2.0 mmol), *p*-toluenesulfonylmethyl isocyanide (390 mg, 2.0 mmol), 3-benzoylacrylic acid (350 mg, 2.0 mmol). The solvent was evaporated *in vacuo* and the residue was purified by column chromatography (1st silica gel 60, dichloromethane/methanol 10:0.5) to give the purified mixture of *cis*- and *trans*-product in a yield of 35%. A second purification step by column chromatography (2nd flash silica gel, chloroform/ethanol 10:0.1) gave the *cis*- and *trans*-diastereomers separately. ***trans*-6c**: Slightly yellow solid (102 mg, 9%): mp 135–138 °C; IR *ν* 2940, 1652, 1596, 1433, 1324, 1141, 1086, 1029, 815, 753, 689 cm^−1^; ^1^H-NMR (400 MHz, DMSO) δ 8.40 (dd, 4.7 Hz, 1.7 Hz, 1H), 8.05 (d, 1.7 Hz, 1H), 8.01 (dd, 7.7 Hz, 1.2 Hz, 2H), 7.71 (d, 8.3 Hz, 2H), 7.69 (tt, 7.7 Hz, 1.2 Hz, 1H), 7.57 (t, 7.7 Hz, 2H), 7.38–7.31 (m, 5H), 7.30–7.25 (m, 1H), 7.23 (dd, 7.8 Hz, 4.7 Hz, 1H), 7.17 (d, 7.0 Hz, 1H), 5.66 (s, 1H), 5.38 (d, 14.8 Hz, 1H), 4.97 (d, 14.8 Hz, 1H), 4.94 (dd, 4.2 Hz, 3.4 Hz, 1H), 4.83 (d, 15.4 Hz, 1H), 4.31 (dd, 19.1 Hz, 4.2 Hz, 1H), 3.92 (dd, 19.1 Hz, 3.4 Hz, 1H), 3.77 (d, 15.4 Hz, 1H), 2.34 (s, 3H); ^13^C-NMR (100 MHz, DMSO): δ 197.6, 165.9, 164.6, 149.1, 148.4, 144.7, 135.8, 135.5, 135.0, 133.7, 133.5, 133.4, 130.9, 130.7, 130.1, 129.8, 129.6, 128.6, 128.07, 128.05, 127.4, 123.1, 62.8, 60.4, 55.9, 45.0, 39.9, 21.0; HRMS (MS-TOF): [M+H]^+^ calcd. for C_32_H_29_ClN_3_O_5_S: 602.1511, found: 602.1510. ***cis*-6c**: slightly yellow solid (103 mg, 9%): mp 79–82 °C; IR *ν* 2934, 1657, 1596, 1423, 1326, 1141, 1085, 1028, 815, 752, 688 cm^−1^; ^1^H-NMR (400 MHz, DMSO) δ 8.48 (dd, 4.7 Hz, 1.7 Hz, 1H), 8.22 (d, 1.7 Hz, 1H), 8.09 (d, 7.5 Hz, 2H), 7.83 (d, 7.8 Hz, 1H), 7.70 (tt, 7.5 Hz, 1.2 Hz, 1H), 7.60 (d, 8.0 Hz, 2H), 7.59 (t, 7.5 Hz, 2H), 7.51 (dt, 7.9 Hz, 1.7 Hz, 1H), 7.44–7.36 (m, 3H), 7.36–7.32 (m, 1H), 7.18 (d, 8.0 Hz, 2H), 5.23 (s, 1H), 5.12 (d, 14.5 Hz, 1H), 5.03 (dd, 4.4 Hz, 4.2 Hz, 1H), 5.00 (d, 14.5 Hz, 1H), 4.88 (d, 15.4 Hz, 1H), 4.19 (dd, 18.7 Hz, 4.4 Hz, 1H), 3.98 (dd, 18.7 Hz, 4.2 Hz, 1H), 3.77 (d, 15.4 Hz, 1H), 2.26 (s, 3H); ^13^C NMR (100 MHz, DMSO): δ 196.9, 165.3, 163.9, 149.1, 148.6, 144.7, 136.1, 135.6, 134.1, 133.8, 133.4, 133.2, 131.0, 130.2, 129.8, 129.6, 129.4, 128.6, 128.3, 128.1, 127.8, 123.3, 63.9, 58.9, 56.0, 45.0, 40.7, 20.9; HRMS (MS-TOF): [M+H]^+^ calcd. for C_32_H_29_ClN_3_O_5_S: 602.1511, found: 602.1513.

*3-(2-Iodophenyl)-6-(2-oxo-2-phenylethyl)-4-(pyridin-3-ylmethyl)-1-(tosylmethyl)piperazine-2,5-dione* (**6d**). Quantities: 2-iodobenzaldehyde (465 mg, 2.0 mmol), 3-(aminomethyl)pyridine (200 µL, 2.0 mmol), *p*-toluenesulfonylmethyl isocyanide (390 mg, 2.0 mmol), 3-benzoylacrylic acid (350 mg, 2.0 mmol). The solvent was evaporated *in vacuo* and the residue was purified by column chromatography (1st silica gel 60, dichloromethane/methanol 10:0.5) to give the purified mixture of *cis*- and *trans*-product in a yield of 45%. A second purification step by column chromatography (2nd flash silica gel, chloroform/ethanol 10:0.2) gave the *cis*- and *trans*-diastereomers separately. ***trans*-6d**: Colorless amorphous solid (210 mg, 15%): mp 101–104 °C; IR *ν* 2929, 1657, 1596, 1580, 1422, 1318, 1140, 1085, 1013, 815, 759, 688 cm^−1^; ^1^H-NMR (400 MHz, DMSO) δ 8.41 (dd, 4.8 Hz, 1.7 Hz, 1H), 8.08 (d, 1.7 Hz, 1H), 8.02 (dd, 7.9 Hz, 1.3 Hz, 2H), 7.81 (dd, 7.7 Hz, 1.4 Hz, 1H), 7.73–7.67 (m, 2H), 7.71 (d, 8.2 Hz, 2H), 7.58 (t, 7.9 Hz, 2H), 7.35 (d, 8.2 Hz, 2H), 7.32 (td, 7.7 Hz, 1.1 Hz, 1H), 7.25 (dd, 7.9 Hz, 4.8 Hz, 1H), 7.07 (td, 7.7 Hz, 1.4 Hz, 1H), 7.03 (dd, 7.7 Hz, 1.1 Hz, 1H), 5.61 (s, 1H), 5.39 (d, 14.7 Hz, 1H), 4.96–4.93 (m, 1H), 4.95 (d, 14.7 Hz, 1H), 4.86 (d, 15.5 Hz, 1H), 4.35 (dd, 19.1 Hz, 4.1 Hz, 1H), 3.91 (dd, 19.1 Hz, 3.2 Hz, 1H), 3.67 (d, 15.5 Hz, 1H), 2.34 (s, 3H); ^13^C-NMR (100 MHz, DMSO): δ 197.7, 165.9, 164.6, 149.1, 148.4, 144.7, 139.4, 138.4, 135.8, 135.6, 134.8, 133.7, 132.2, 130.8, 130.2, 129.8, 129.4, 128.6, 128.1 (3C), 101.7, 66.4, 62.5, 55.9, 44.9, 38.8, 21.0; HRMS (MS-TOF): [M+H]^+^ calcd. for C_32_H_29_IN_3_O_5_S: 694.0867, found: 694.0864. ***cis*-6d**: Colorless amorphous solid (75 mg, 5%): mp 115–118 °C; IR *ν* 2933, 1657, 1596, 1579, 1421, 1323, 1140, 1085, 1012, 814, 754, 688 cm^−1^; ^1^H-NMR (400 MHz, DMSO) δ 8.47 (dd, 4.8 Hz, 1.9 Hz, 1H), 8.26 (d, 1.9 Hz, 1H), 8.10 (dd, 7.7 Hz, 1.2 Hz, 2H), 7.82 (dd, 7.8 Hz, 1.1 Hz, 1H), 7.78 (dd, 7.8 Hz, 1.3 Hz, 1H), 7.70 (tt, 7.7 Hz, 1.2 Hz, 1H), 7.62–7.57 (m, 4H), 7.52 (dt, 8.0 Hz, 1.9 Hz, 1H), 7.45 (td, 7.8 Hz, 1.1 Hz, 1H), 7.35 (dd, 8.0 Hz, 4.8 Hz, 1H), 7.20 (d, 8.0 Hz, 2H), 7.08 (td, 7.8 Hz, 1.3 Hz, 1H), 5.14 (s, 1H), 5.11 (d, 14.5 Hz, 1H), 5.04 (dd, 4.4 Hz, 3.9 Hz, 1H), 5.00 (d, 14.5 Hz, 1H), 4.85 (d, 15.4 Hz, 1H), 4.19 (dd, 18.9 Hz, 4.4 Hz, 1H), 3.99 (dd, 18.6 Hz, 3.9 Hz, 1H), 3.73 (d, 15.4 Hz, 1H), 2.27 (s, 3H); ^13^C-NMR (100 MHz, DMSO): δ 196.9, 165.2, 164.0, 149.0, 148.5, 144.8, 139.4, 138.3, 136.1, 135.6, 134.0, 133.4, 131.1, 130.3, 129.7, 129.0, 128.8, 128.6, 128.3, 128.1, 123.4, 102.5, 66.0, 63.9, 55.9, 44.9, 40.8, 21.1; HRMS (MS-TOF): [M+H]^+^ calcd. for C_32_H_29_IN_3_O_5_S: 694.0867, found: 694.0868.

*3-(4-Fluorophenyl)-6-(2-oxo-2-phenylethyl)-4-(pyridin-3-ylmethyl)-1-(tosylmethyl)piperazine-2,5-dione* (***trans*-6e**). Quantities: 4-fluorobenzaldehyde (220 µL, 2.0 mmol), 3-(aminomethyl)pyridine (200 µL, 2.0 mmol), *p*-toluenesulfonylmethyl isocyanide (390 mg, 2.0 mmol), 3-benzoylacrylic acid (350 mg, 2.0 mmol). The precipitate was filtered and washed with cold methanol to give pure ***trans*-6e**. Colorless solid (494 mg, 42%): mp 174–175 °C; IR *ν* 2984, 1684, 1652, 1597, 1579, 1510, 1422, 1329, 1221, 1145, 1088, 812, 753, 687 cm^−1^; ^1^H-NMR (400 MHz, DMSO) δ 8.41 (dd, 4.9 Hz, 1.8 Hz, 1H), 8.17 (d, 1.8 Hz, 1H), 7.98 (d, 7.7 Hz, 2H), 7.69 (tt, 7.7 Hz, 1.1 Hz, 1H), 7.65 (d, 8.3 Hz, 2H), 7.57 (t, 7.7 Hz, 2H), 7.41 (dt, 8.0 Hz, 1.8 Hz, 1H), 7.28 (d, 8.0 Hz, 2H), 7.25 (dd, 8.0 Hz, 4.9 Hz, 1H), 7.12 (d, 7.3 Hz, 4H), 5.49 (d, 14.9 Hz, 1H), 5.24 (s, 1H), 4.93 (dd, 4.2 Hz, 3.5 Hz, 1H), 4.88 (d, 14.9 Hz, 1H), 4.82 (d, 15.3 Hz, 1H), 4.25 (dd, 18.9 Hz, 4.2 Hz, 1H), 3.89 (dd, 18.9 Hz, 3.5 Hz, 1H), 3.84 (d, 15.3 Hz, 1H), 2.34 (s, 3H); ^13^C-NMR (100 MHz, DMSO): δ 197.4, 165.6, 165.3, 161.9 (d, ^1^*J*(C,F) = 244.9 Hz), 149.0, 148.3, 144.7, 135.9, 135.5, 134.5, 133.6, 132.1 (d, ^4^*J*(C,F) = 3.0 Hz), 131.4, 130.2 (d, ^3^*J*(C,F) = 8.6 Hz), 129.7, 128.6, 128.0 (2C), 123.1, 115.2 (d, ^2^*J*(C,F) = 21.9 Hz), 62.7, 61.5, 55.4, 45.2, 38.0, 20.9; LCMS (ESI^+^): *m*/*z* 586.0 [M+H]^+^; Anal. calcd. for C_32_H_28_FN_3_O_5_S: C 65.63, H 4.82, N 7.18, S 5.48; found: C 65.95, H 4.88, N 7.27, S 5.36.

*1-Benzyl-3-(2-oxo-2-phenylethyl)-6-phenyl-4-(tosylmethyl)piperazine-2,5-dione* (***trans*-6f**). Quantities: benzaldehyde (200 µL, 2.0 mmol), benzylamine (220 µL, 2.0 mmol), *p*-toluenesulfonylmethyl isocyanide (390 mg, 2.0 mmol), 3-benzoylacrylic acid (350 mg, 2.0 mmol). The precipitate was filtered and washed with cold methanol to give pure ***trans*-6f**. Colorless solid (628 mg, 55%): mp 201–203 °C; IR *ν* 2929, 1662, 1598, 1421, 1318, 1143, 1087, 814, 749, 691 cm^−1^; ^1^H-NMR (400 MHz, DMSO) δ 8.01 (dd, 7.4 Hz, 1.5 Hz, 2H), 7.69 (tt, 7.4 Hz, 1.5 Hz, 1H), 7.62 (d, 8.3 Hz, 2H), 7.58 (t, 7.4 Hz, 2H), 7.41–7.31 (m, 3H), 7.27–7.22 (m, 5H), 7.05 (dd, 7.8 Hz, 1.4 Hz, 2H), 7.00 (dd, 6.6 Hz, 2.9 Hz, 2H), 5.48 (d, 14.9 Hz, 1H), 5.05 (d, 15.2 Hz, 1H), 5.04 (s, 1H), 4.92 (t, 3.7 Hz, 1H), 4.87 (d, 14.9 Hz, 1H), 4.20 (dd, 18.8 Hz, 3.7 Hz, 1H), 3.90 (dd, 18.8 Hz, 3.7 Hz, 1H), 3.57 (d, 15.2 Hz, 1H), 2.34 (s, 3H); ^13^C NMR (100 MHz, DMSO): δ 197.2, 165.5, 165.4, 144.7, 136.0, 135.5, 135.4, 134.5, 133.6, 129.7, 128.6, 128.5, 128.3, 128.2, 128.02, 127.97, 127.7 (2C), 127.2, 62.9, 61.5, 55.4, 46.9, 37.7, 21.0; LCMS (ESI^+^): *m*/*z* 566.8 [M]^+^; Anal. calcd. for C_33_H_30_N_2_O_5_S: C 69.94, H 5.34, N 4.94, S 5.66; found: C 70.10, H 5.35, N 5.07, S 5.60.

*3-(2-Oxo-2-phenylethyl)-1-phenethyl-6-phenyl-4-(tosylmethyl)piperazine-2,5-dione* (***trans*-6g**). Quantities: benzaldehyde (200 µL, 2.0 mmol), phenethylamine (250 µL, 2.0 mmol), *p*-toluenesulfonylmethyl isocyanide (390 mg, 2.0 mmol), 3-benzoylacrylic acid (350 mg, 2.0 mmol). The precipitate was filtered, dissolved in dichloromethane and extracted with aqueous saturated solution of sodium hydrogen carbonate to give pure ***trans*-6g**. Colorless solid (135 mg, 12%): mp 198–201 °C; IR *ν* 2931, 1685, 1655, 1597, 1427, 1326, 1142, 1087, 813, 752, 689 cm^−1^; ^1^H-NMR (400 MHz, DMSO) δ 8.02 (dd, 7.5 Hz, 1.3 Hz, 2H), 7.69 (tt, 7.5 Hz, 1.3 Hz, 1H), 7.65 (d, 8.2 Hz, 2H), 7.57 (t, 7.5 Hz, 2H), 7.39–7.34 (m, 3H), 7.31 (d, 8.2 Hz, 2H), 7.21 (t, 7.2 Hz, 2H), 7.17 (dd, 7.1 Hz, 2.2 Hz, 2H), 7.14 (tt, 7.2 Hz, 1.4 Hz, 1H), 7.00 (dd, 7.2 Hz, 1.4 Hz, 2H), 5.49 (d, 14.8 Hz, 1H), 5.36 (s, 1H), 4.88 (d, 14.8 Hz, 1H), 4.84 (dd, 4.4 Hz, 3.6 Hz, 1H), 4.15 (dd, 18.7 Hz, 4.4 Hz, 1H), 3.83 (dd, 18.7 Hz, 3.6 Hz, 1H), 3.71 (ddd, 13.3 Hz, 10.7 Hz, 5.3 Hz, 1H), 2.80 (ddd, 12.7 Hz, 10.5 Hz, 5.3 Hz, 1H), 2.65 (ddd, 13.3 Hz, 10.5 Hz, 5.6 Hz, 1H), 2.40 (ddd, 12.7 Hz, 10.7 Hz, 5.6 Hz, 1H), 2.36 (s, 3H); ^13^C-NMR (100 MHz, DMSO): δ 197.1, 165.8, 164.9, 144.7, 138.5, 136.6, 135.9, 134.6, 133.6, 129.8, 128.6, 128.5, 128.33, 128.28, 128.2, 128.1, 128.0 (2C), 126.1, 63.2, 61.8, 55.4, 46.1, 38.1, 31.6, 21.0; Anal. calcd. for C_34_H_32_N_2_O_5_S: C 70.32, H 5.55, N 4.82, S 5.52; found: C 70.44, H 5.68, N 4.93, S 5.52.

*1-(4-Methoxybenzyl)-3-(2-oxo-2-phenylethyl)-6-phenyl-4-(tosylmethyl)piperazine-2,5-dione* (***trans*-6h**). Quantities: benzaldehyde (200 µL, 2.0 mmol), 4-methoxybenzylamine (260 µL, 2.0 mmol), *p*-toluenesulfonylmethyl isocyanide (390 mg, 2.0 mmol), 3-benzoylacrylic acid (350 mg, 2.0 mmol). The precipitate was filtered and washed with cold methanol to give pure ***trans*-6h**. Colorless solid (460 mg, 39%): mp 241–243 °C; IR *ν* 2930, 1659, 1611, 1598, 1611, 1598, 1510, 1432, 1318, 1247, 1178, 1144, 1088, 824, 754, 690 cm^−1^; ^1^H-NMR (400 MHz, DMSO) δ 8.00 (dd, 7.6 Hz, 1.2 Hz, 2H), 7.69 (tt, 7.6 Hz, 1.2 Hz, 1H), 7.60 (d, 8.1 Hz, 2H), 7.58 (t, 7.6 Hz, 2H), 7.42–7.33 (m, 3H), 7.24 (d, 8.1 Hz, 2 H), 7.05 (dd, 7.7 Hz, 1.6 Hz, 2H), 6.92 (d, 8.7 Hz, 2H), 6.80 (d, 8.7 Hz, 2H), 5.47 (d, 14.9 Hz, 1H), 5.04 (d, 14.9 Hz, 1H), 4.98 (s, 1H), 4.90 (dd, 4.5 Hz, 3.7 Hz, 1H), 4.86 (d, 14.9 Hz, 1H), 4.18 (dd, 18.7 Hz, 4.5 Hz, 1H), 3.88 (dd, 18.7 Hz, 3.7 Hz, 1H), 3.72 (s, 3H), 3.44 (d, 14.9 Hz, 1H), 2.33 (s, 3H); ^13^C-NMR (100 MHz, DMSO): δ 197.1, 165.6, 165.3, 158.5, 144.6, 136.0, 135.6, 134.5, 133.5, 129.7, 129.3, 128.6, 128.5, 128.2, 128.02, 127.96, 127.7, 127.1, 113.6, 62.6, 61.5, 55.4, 54.9, 46.2, 37.6, 21.0; LCMS (ESI^+^): *m*/*z* 596.4 [M]^+^; Anal. calcd. for C_34_H_32_N_2_O_6_S: C 68.44, H 5.41, N 4.69, S 5.37; found: C 68.47, H 5.43, N 4.86, S 5.40.

*3-(2-(4-Chlorophenyl)-2-oxoethyl)-6-phenyl-1-(pyridin-3-ylmethyl)-4-(tosylmethyl)piperazine-2,5-dione* (***trans*-6i**). Quantities: benzaldehyde (200 µL, 2.0 mmol), 3-(aminomethyl)pyridine (200 µL, 2.0 mmol), *p*-toluenesulfonylmethyl isocyanide (390 mg, 2.0 mmol), 3-(4-chlorobenzoyl)acrylic acid (420 mg, 2.0 mmol). The precipitate was filtered, dissolved in ethyl acetate and crystallization initiated by adding *n*-hexane to give pure ***trans*-6i**. Colorless solid (449 mg, 37%): mp 233–234 °C; IR *ν* 2940, 1686, 1651, 1591, 1576, 1435, 1325, 1142, 815, 754, 699 cm^−1^; ^1^H-NMR (400 MHz, DMSO) δ 8.46 (dd, 4.7 Hz, 1.8 Hz, 1H), 8.17 (d, 1.8 Hz, 1H), 7.99 (ddd, 8.6 Hz, 2.4 Hz, 1.8 Hz, 2H), 7.64 (ddd, 8.8 Hz, 2.4 Hz, 1.8 Hz, 2H), 7.62 (d, 8.5 Hz, 2H), 7.41 (dt, 7.9 Hz, 1.8 Hz, 1H), 7.37–7.29 (m, 3H), 7.26 (d, 8.5 Hz, 2H), 7.26–7.23 (m, 1H), 7.07 (dd, 7.8 Hz, 1.1 Hz, 2H), 5.46 (d, 14.9 Hz, 1H), 5.18 (s, 1H), 4.93 (dd, 4.4 Hz, 3.6 Hz, 1H), 4.88 (d, 14.9 Hz, 1H), 4.86 (d, 15.3 Hz, 1H), 4.21 (dd, 18.9 Hz, 4.4 Hz, 1H), 3.88 (dd, 18.9 Hz, 3.6 Hz, 1H), 3.79 (d, 15.3 Hz, 1H), 2.34 (s, 3H); ^13^C-NMR (100 MHz, DMSO): δ 196.4, 165.6, 165.4, 149.0, 148.3, 144.7, 128.7, 138.5, 135.6, 135.4, 134.6, 134.5, 131.4, 129.9, 129.7, 128.5, 128.3, 127.98, 127.92, 123.2, 63.4, 61.7, 55.4, 45.3, 38.0, 21.0; LCMS (ESI^+^): *m*/*z* 602.0 [M]^+^; HRMS (MS-TOF): [M+H]^+^ calcd. for C_32_H_29_ClN_3_O_5_S: 602.1511, found: 602.1515.

*3-(2-(4-Methoxyphenyl)-2-oxoethyl)-6-phenyl-1-(pyridin-3-ylmethyl)-4-(tosylmethyl)piperazine-2,5-dione* (***trans*-6j**). Quantities: benzaldehyde (200 µL, 2.0 mmol), 3-(aminomethyl)pyridine (200 µL, 2.0 mmol), *p*-toluenesulfonylmethyl isocyanide (390 mg, 2.0 mmol), 3-(4-methoxybenzoyl)acrylic acid (410 mg, 2.0 mmol). The precipitate was filtered and washed with cold methanol to give pure ***trans*-6j**. Colorless solid (434 mg, 36%): mp 250–252 °C; IR *ν* 2938, 1650, 1601, 1576, 1510, 1437, 1327, 1261, 1171, 1142, 1089, 825, 754, 699 cm^−1^; ^1^H-NMR (400 MHz, DMSO) δ 8.41 (dd, 4.8 Hz, 1.8 Hz, 1H), 8.15 (d, 1.8 Hz, 1H), 7.97 (d, 9.0 Hz, 2H), 7.64 (d, 8.2 Hz, 2H), 7.39 (dt, 7.9 Hz, 1. 8 Hz, 1H), 7.36-7.30 (m, 3H), 7.24 (dd, 7.9 Hz, 4.8 Hz, 1H), 7.28 (d, 8.2 Hz, 2H), 7.08 (d, 9.0 Hz, 2H), 7.06 (dd, 8.3 Hz, 1.4 Hz, 2H), 5.48 (d, 14.8 Hz, 1H), 5.17 (s, 1H), 4.89 (dd, 4.3 Hz, 3.2 Hz, 1H), 4.85 (d, 15.4 Hz, 1H), 4.84 (d, 14.8 Hz, 1H), 4.18 (18.7 Hz, 4.3 Hz, 1H), 3.87 (s, 3H), 3.81 (18.7 Hz, 3.2 Hz, 1H), 3.78 (d, 15.4 Hz, 1H), 2.34 (s, 3H); ^13^C NMR (100 M Hz, DMSO): δ 195.6, 165.8, 165.6, 163.5, 149.0, 148.3, 144.7, 136.0, 135.4, 134.6, 131.4, 130.4, 129.8, 128.9, 128.4, 128.3, 128.04, 128.00, 123.1, 113.8, 63.4, 61.6, 55.52, 55.50, 45.2, 37.5, 21.0; LCMS (ESI^+^): *m*/*z* 598.2 [M+H]^+^; HRMS (MS-TOF): [M+H]^+^ calcd. for C_33_H_32_N_3_O_6_S: 598.2006, found: 598.2009.

### 3.4. Bioactivity Analyses

#### 3.4.1. Isolation and Activation of T Cells

T cells were isolated from the peripheral blood of human healthy donors by Biocoll density gradient centrifugation (Biochrom, Berlin, Germany) and subsequent positive isolation using magnetic cell sorting techniques (Miltenyi Biotec, Bergisch-Gladbach, Germany). For activation, T cells were cocultured for 5 days with allogeneic dendritic cells (DCs) in culture medium supplemented with 10% heat-inactivated, pooled human serum and 100 U/mL penicillin-streptomycin (Invitrogen, Darmstadt, Germany). DCs were generated within 3 days from isolated monocytes according to a previously described protocol [24] and matured by addition of 25 ng/mL tumor necrosis factor-α, 5 ng/mL IL-1β, 10 ng/mL IL-6 (all R&D Systems, Wiesbaden, Germany), and 1 µg/mL prostaglandin E2 (Sigma-Aldrich, Munich, Germany) for 24 h to the culture medium. Newly synthesized compounds were added at day 4 to the activation culture for the last 24 h.

#### 3.4.2. Analysis of T Cell Proliferation

T cell proliferation was quantified by measuring [^3^H]thymidine (GE healthcare, Munich, Germany) incorporation. Synthesized compounds and 17-DMAG (Invivogen, Toulouse, France) were resolved in DMSO (Sigma-Aldrich, Munich, Germany). Activated T cells were incubated on day 4 for the last 24 h with the indicated concentrations of synthesized compounds, 17-DMAG or DMSO control and pulsed with 5µCi/mL [^3^H]thymidine for the last 18 h. Incorporation of [^3^H]thymidine was quantified using a beta-counter (Perkin-Elmer, Rodgau, Germany).

#### 3.4.3. Analysis of Apoptosis

Apoptotic cell death was determined using AnnexinV-Cy5/7-AAD staining. Briefly, resting T cells were treated for 24 h with the indicated concentrations of the synthesized compounds, 17-DMAG or DMSO ctrl washed with phosphate-buffered saline and resuspended in binding buffer in a concentration of 1 × 10^6^ cells/mL. Annexin V-Cy5 (BD biosciences, Heidelberg, Germany) and 7-AAD (ebioscience, Frankfurt, Germany) were added and cells were incubated for 15 min at room temperature in the dark. Apoptosis of cells was measured and quantified using flow cytometry. Percentages of specific apoptosis (sA) were calculated as follows:

$$sA\;(\%)=100\times \frac{A_{E}-A_{C}}{100-A_{C}}$$

where A_E_ equals % of apoptotic cells in the experimental group, and A_C_ equals % of apoptotic cells in the control group.

#### 3.4.4. Statistical Analyses

Statistical analyses were performed using Microsoft EXCEL software (Munich, Germany). Differences between mean values were assessed using two-tailed paired Student’s *t*-test. Statistical significance was set at *p* < 0.05.

### 3.5. X-ray Crystallography

The single-crystals of compound **6b** were obtained by slow diffusion of petroleum ether to a compound solution in dichloromethane. Intensity data were collected at a Temperature of 173 K on a Bruker APEX-CCD (D8 three-circle goniometer) (Bruker AXS) diffractometer with graphite monochromated Mo-K*α* radiation (*λ* = 0.71073 Å) using the *ω* scan mode with 1.74° < *θ* < 25.00°; 20749 reflections were measured with 4764 unique reflections. 3326 reflections with *I* > 2*σ*(*I*) were used in the Fourier techniques. The final refinement was converged to *R*1 = 0.0919 [*I* > 2σ(*I*)], *wR*2(*F_o_^2^*) = 0.2499 (all data). Crystal data: empirical formula C_32_H_29_N_3_O_5_S, crystal dimension 0.24 × 0.06 × 0.05 mm, monoclinic, space group *P*2_1_/n, *a* = 5.7974(8) Å, *b* = 23.460(3) Å, *c* = 20.101(3) Å, *β* = 96.219(3) °, V = 2717.8(6) Å^3^, *M*r = 567.64, Z = 4, *D*c = 1.387 Mg/m^3^, *μ*(Mo K*α*) = 0.168 mm^−1^, *F*(000) = 1192, *S* = 1.096. Crystallographic data (excluding structure factors) have been deposited with the Cambridge Crystallographic Data Centre as supplementary publication No. CCDC-910534. Copies of the data can be obtained free of charge on application to Cambridge Crystallographic Data Centre, 12 Union Road, Cambridge CB2 1EZ, UK; [fax: (+44)-1223-336-033; email: deposit@ccdc.cam.ac.uk]. Details of the crystal structure are given in the crystallographic figure and data tables (Supplementary Figure 1, Tables 2 and 3).

## 4. Conclusions

In summary, we were able to synthesize a library of differently substituted 2,5-diketopiperazines via a coupled Ugi/Aza-Michael reaction by using β-acyl substituted acrylic acids. The stereochemistry of the highly substituted ring system was confirmed by X-ray crystallography and NMR spectroscopy. The small library of 2,5-diketopiperazines was analysed with respect to their anti-proliferative properties. Proliferation analyses using activated human T cells demonstrated a significant anti-proliferative effect of the 2,5-diketopiperazine ***trans*-6a**, ***trans*-6c**, ***trans*-6d** in noncytotoxic concentrations. Detailed experiments analysing apoptosis and cell cycle in activated T cells to explore the underlying mechanism of the observed anti-proliferative effect are in progress.

## Figures and Tables

**Table 1:**
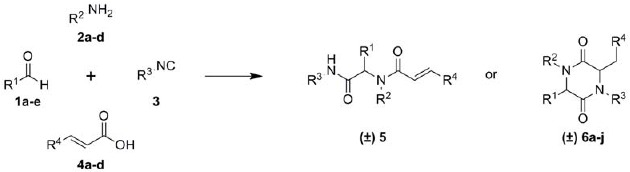
Synthesized acyclic Ugi product 5 and 2,5-diketopiperazines **6a**–**j**.

#	R^1^	R^2^	R^3^	R^4^	product	*cis*:*trans*	Yield [%]
1	2-Br-Ph (**1a**)	pyridin-3ylmethyl (**2a**)	tosylmethyl (**3**)	Ph (**4a**)	(±) **5**	-	17
2	2-Br-Ph (**1a**)	pyridin-3ylmethyl (**2a**)	tosylmethyl (**3**)	benzoyl (**4b**)	(±) **6a**	1.6:1	35 ^b^
3	Ph (**1b**)	pyridin-3ylmethyl (**2a**)	tosylmethyl (**3**)	benzoyl (**4b**)	(±) **6b**	*trans* ^a^	45
4	2-Cl-Ph (**1c**)	pyridin-3ylmethyl (**2a**)	tosylmethyl (**3**)	benzoyl (**4b**)	(±) **6c**	2.2:1	35 ^b^
5	2-I-Ph (**1d**)	pyridin-3ylmethyl (**2a**)	tosylmethyl (**3**)	benzoyl (**4b**)	(±) **6d**	1:1	43 ^b^
6	4-F-Ph (**1e**)	pyridin-3ylmethyl (**2a**)	tosylmethyl (**3**)	benzoyl (**4b**)	(±) **6e**	*trans* ^a^	42
7	Ph (**1b**)	Bn (**2b**)	tosylmethyl (**3**)	benzoyl (**4b**)	(±) **6f**	*trans* ^a^	55
8	Ph (**1b**)	phenethyl (**2c**)	tosylmethyl (**3**)	benzoyl (**4b**)	(±) **6g**	*trans* ^a^	12
9	Ph (**1b**)	4-MeO-Bn (**2d**)	tosylmethyl (**3**)	benzoyl (**4b**)	(±) **6h**	*trans* ^a^	39
10	Ph (**1b**)	pyridin-3ylmethyl (**2a**)	tosylmethyl (**3**)	4-Cl-benzoyl (**4c**)	(±) **6i**	*trans* ^a^	37
11	Ph (**1b**)	pyridin-3ylmethyl (**2a**)	tosylmethyl (**3**)	4-MeO-benzoyl (**4d**)	(±) **6j**	*trans* ^a^	36

^a^ Precipitation of the pure *trans* product; ^b^ Yields of the purely isolated diastereomeric mixture is reported.

## Supplementary Materials

Supplementary materials can be accessed at: http://www.mdpi.com/1420-3049/17/12/14685/s1.
