# Supplementary material for: One-Pot Ugi/Aza-Michael Synthesis of Highly Substituted 2,5-Diketopiperazines with Anti-Proliferative Properties
Source: Molecules. 2012 Dec 11;17(12):14685–99. doi: 10.3390/molecules171214685 (PMC6268348; doi:10.3390/molecules171214685)
Supplement: Supplementary file 1 [file molecules-17-14685-s001.pdf]

Supporting Information

One-Pot Ugi/Aza-Michael Synthesis of Highly Substituted 2,5-Diketopiperazines with Anti-proliferative Properties

Andreas Hartung, Florian Seufert, Carsten Berges, Viktoria H. Gessner and Ulrike Holzgrabe

Table of Contents:

- I. Proliferation and Cytotoxity data of synthesized 2,5-DKPs: page S2
- II. Crystal Structure Data: page S3–S5

I. Proliferation and Cytotoxity data of synthesized 2,5-DKPs

Table 1. Effect of 17-DMAG and the synthesized 2,5-DKPs on T cells.

Comp	Conc [µM]	Effect on Proliferation [a,b]	Cytotoxity [c,d]
**DMAG**	0.5	54.3 (3.0)	-
	2	41.6 (2.6)	-
	5	43.9 (1.7)	2.9 (1.8)
	10	37.0 (2.2)	9.9 (3.1)
***cis*-6a**	1	92.9 (2.5)	-
	5	100.2 (15.3)	-
	10	86.2 (14.2)	-
	20	67.8 (8.3)	0.4 (0.9)
	50	44.8 (6.1)	11.7 (6.4)
***trans*-6a**	1	86.4 (16.0)	-
	5	59.5 (8.3)	-
	10	43.3 (4.3)	-
	20	24.0 (2.3)	-
	50	11.0 (1.3)	2.4 (1.2)
***trans*-6b**	10	64.2 (6.7)	-
	20	54.8 (3.2)	-
	50	30.0 (2.7)	1.7 (0.3)
***cis*-6c**	1	94.9 (11.3)	-
	5	86.2 (9.8)	-
	10	68.6 (11.5)	-
	20	62.0 (5.1)	-
	50	35.6 (4.8)	4.8 (1.2)
***trans*-6c**	1	80.4 (11.9)	-
	5	61.7 (8.5)	-
	10	41.5 (5.8)	-
	20	28.2 (4.8)	0.2 (0.2)
	50	14.8 (3.2)	3.9 (1.7)
***cis*-6d**	1	101.6 (12.1)	-
	5	80.8 (3.3)	-
	10	75.1 (9.1)	-
	20	58.8 (7.6)	-
	50	48.5 (7.4)	1.7 (1.1)
***trans*-6d**	1	87.8 (18.0)	-
	5	60.7 (15.3)	-
	10	44.3 (17.4)	-
	20	34.1 (14.8)	-
	50	25.6 (11.3)	-
***trans*-6e**	10	68.1 (6.4)	-
	20	79.1 (6.3)	-
	50	63.7 (8.0)	-
***trans*-6f**	10	92.1 (8.5)	-
	20	93.4 (9.7)	-
	50	68.6 (3.5)	-
***trans*-6g**	10	66.4 (2.5)	7.0 (1.3)
	20	71.4 (4.4)	12.5 (2.3)
	50	74.2 (9.5)	14.0 (4.2)
***trans*-6h**	10	58.7 (5.0)	-
	20	49.7 (4.2)	-
	50	47.5 (2.9)	-
***trans*-6i**	10	29.9 (1.6)	20.2 (7.1)
	20	19.3 (2.6)	46.0 (2.2)
	50	48.5 (7.4)	30.4 (7.2)
***trans*-6j**	10	98.1 (9.2)	-
	20	89.1 (8.0)	-
	50	77.4 (6.5)	0.2 (1.0)

[a] [^{3}H]-thymidine uptake [% of DMSO ctrl] (SEM); [b] Data represent mean values of at least 4 independent experiments carried out in triplicate; [c] Specific apoptosis in resting T cells [%] (SEM)); [d] Data are given as mean values of at least 4 independent experiments carried out in duplicate.

II. Crystal Structure Data

Crystal Structure Determination of Compounds 6b

Data collection of all compounds was conducted with a Bruker APEX-CCD (D8 three-circle goniometer) (Bruker AXS), cell determination and –refinement with Smart version 5.622 (Bruker AXS, 2001), integration with SaintPlus version 7.53); empirical absorption correction with Sadabs version 2.10. The crystal was mounted in an inert oil (perfluoropolyalkylether) at −60 °C (N_2 stream), structure determinations were effected at −100 °C (type of radiation: Mo-Kα, λ = 0.71073 Å). The structures were solved applying direct and fourier methods, using SHELXS-90 (G. M. Sheldrick, University of Göttingen 1990) and SHELXL-97 (G. M. Sheldrick, SHELXL97, University of Göttingen 1997).

Figure 1. ORTEP plot of **6b** at 50% probability level. Selected bond lengths [Å] and angles [°]: C(2)-O(1) 1.223(6), C(3)-N(2) 1.452(6), C(3)-C(22) 1.522(7), C(5)-N(2) 1.340(7), C(5)-O(2) 1.213(6), C(5)-C(6) 1.525(7), C(6)-N(1) 1.473(6); C(2)-N(1)-C(6) 123.7(4), C(5)-N(2)-C(3) 124.9(4), N(1)-C(6)-C(5) 114.2(4), N(1)-C(2)-C(3) 118.9(4).

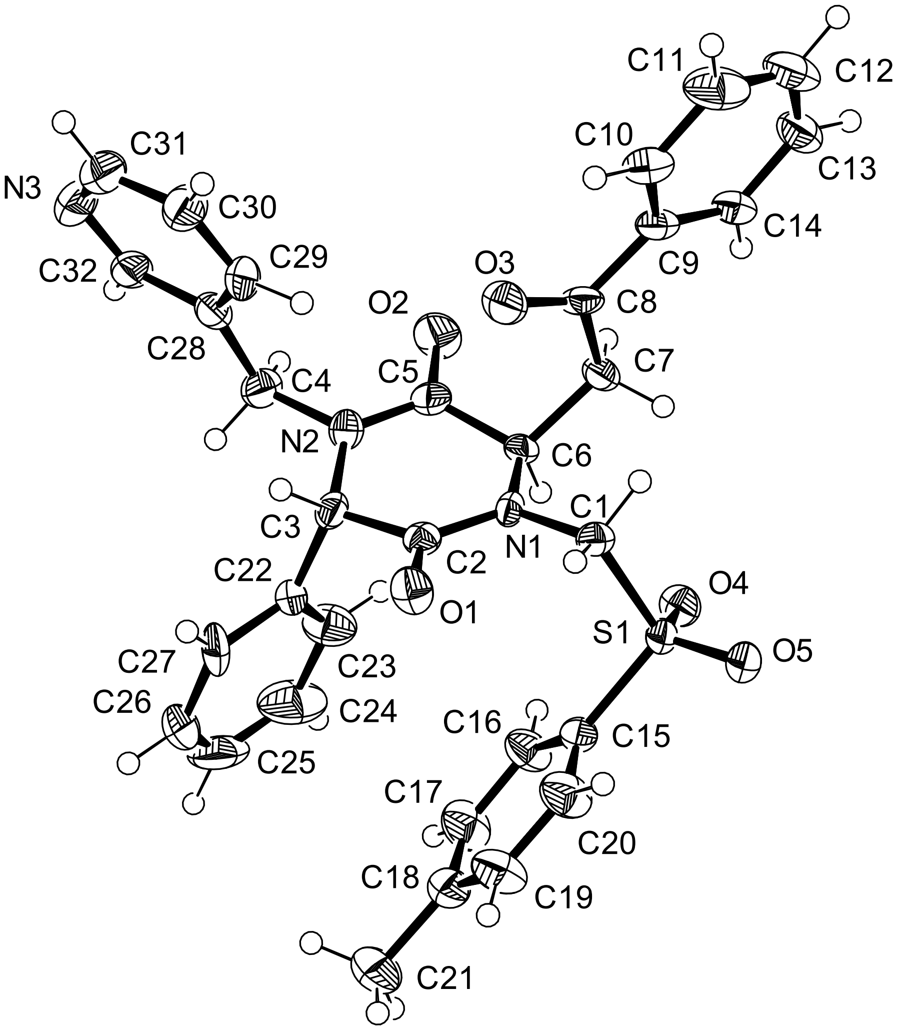

Table 2. Atomic coordinates (×10^4) and equivalent isotropic displacement parameters (Å^2 × 10^3) for **6b**. U(eq) is defined as one third of the trace of the orthogonalized U^{ij} tensor.

	x	y	z	U(eq)
C(1)	8984(9)	727(2)	5589(2)	27(1)
C(2)	9597(9)	1583(2)	6208(2)	26(1)
C(3)	8807(9)	2164(2)	6429(3)	28(1)
C(4)	6472(11)	3014(2)	6082(3)	39(1)
C(5)	5538(10)	2124(2)	5530(3)	32(1)
C(6)	5878(9)	1482(2)	5472(2)	26(1)
C(7)	5377(9)	1313(2)	4732(2)	30(1)
C(8)	7130(9)	1563(2)	4309(3)	30(1)
C(9)	6975(9)	1407(2)	3588(2)	30(1)
C(10)	8826(10)	1554(2)	3239(3)	38(1)
C(11)	8698(13)	1442(3)	2559(3)	55(2)
C(12)	6766(12)	1187(3)	2231(3)	50(2)
C(13)	4950(11)	1030(3)	2576(3)	43(2)
C(14)	5051(10)	1135(2)	3256(3)	34(1)
C(15)	8424(10)	245(2)	6848(2)	31(1)
C(16)	6881(11)	497(3)	7236(3)	40(1)
C(17)	7561(12)	568(3)	7904(3)	49(2)
C(18)	9726(11)	408(2)	8196(3)	40(1)
C(19)	11223(11)	163(3)	7792(3)	48(2)
C(20)	10599(11)	76(3)	7120(3)	44(2)
C(21)	10453(14)	505(3)	8930(3)	60(2)
C(22)	8246(10)	2143(2)	7151(3)	30(1)
C(23)	6121(11)	1951(3)	7303(3)	49(2)
C(24)	5676(15)	1934(3)	7971(4)	71(2)
C(25)	7309(19)	2104(3)	8474(3)	73(3)
C(26)	9390(18)	2284(3)	8311(3)	71(2)
C(27)	9904(13)	2308(3)	7657(3)	51(2)
C(28)	7915(9)	3422(2)	5725(3)	31(1)
C(29)	9626(11)	3260(2)	5342(3)	38(1)
C(30)	10965(12)	3680(2)	5079(3)	45(2)
C(31)	10487(13)	4242(3)	5196(3)	50(2)
C(32)	7556(11)	4002(2)	5807(3)	38(1)
N(1)	8158(7)	1276(2)	5771(2)	23(1)
N(2)	6948(7)	2415(2)	5981(2)	28(1)
N(3)	8806(10)	4412(2)	5556(3)	52(1)
O(1)	11494(6)	1407(2)	6447(2)	37(1)
O(2)	3949(7)	2349(2)	5185(2)	49(1)
O(3)	8606(7)	1891(2)	4551(2)	46(1)
O(4)	5166(6)	203(2)	5849(2)	36(1)
O(5)	8727(7)	-365(2)	5773(2)	35(1)
S(1)	7636(2)	144(1)	5982(1)	27(1)

Table 3. Anisotropic displacement parameters (Å^2 × 10^3) for **6b**. The anisotropic displacement factor exponent takes the form: $-2\pi^2[h^2a^{*2}U^{11} + ... + 2 h k a^* b^* U^{12}]$.

	U^{11}	U^{22}	U^{33}	U^{23}	U^{13}	U^{12}
C(1)	30(3)	27(3)	23(3)	2(2)	3(2)	−2(2)
C(2)	26(3)	28(3)	23(3)	4(2)	8(2)	−2(2)
C(3)	31(3)	22(2)	31(3)	0(2)	1(2)	−5(2)
C(4)	54(4)	31(3)	33(3)	−3(2)	15(3)	6(3)
C(5)	35(3)	35(3)	27(3)	6(2)	7(2)	−1(2)
C(6)	30(3)	29(3)	19(2)	2(2)	3(2)	−5(2)
C(7)	34(3)	34(3)	22(3)	1(2)	2(2)	−5(2)
C(8)	32(3)	32(3)	26(3)	13(2)	−4(2)	−1(2)
C(9)	33(3)	33(3)	23(3)	10(2)	5(2)	3(2)
C(10)	37(3)	43(3)	34(3)	12(2)	6(3)	2(3)
C(11)	57(4)	70(5)	41(4)	16(3)	23(3)	15(4)
C(12)	61(5)	64(4)	25(3)	6(3)	4(3)	17(4)
C(13)	52(4)	48(4)	26(3)	−2(3)	−4(3)	12(3)
C(14)	36(3)	39(3)	25(3)	6(2)	0(2)	3(3)
C(15)	41(3)	29(3)	23(3)	3(2)	7(2)	−2(2)
C(16)	39(3)	53(4)	28(3)	−4(3)	4(3)	10(3)
C(17)	55(4)	60(4)	35(3)	−12(3)	15(3)	11(3)
C(18)	54(4)	36(3)	30(3)	0(2)	3(3)	3(3)
C(19)	45(4)	61(4)	35(3)	2(3)	−2(3)	9(3)
C(20)	46(4)	54(4)	33(3)	−4(3)	7(3)	15(3)
C(21)	91(6)	61(4)	28(3)	−5(3)	3(3)	4(4)
C(22)	43(3)	23(2)	24(3)	−2(2)	1(2)	0(2)
C(23)	46(4)	63(4)	39(4)	8(3)	11(3)	−3(3)
C(24)	81(6)	85(6)	52(5)	24(4)	33(4)	18(5)
C(25)	142(9)	50(4)	30(4)	15(3)	24(5)	29(5)
C(26)	138(8)	44(4)	28(4)	−10(3)	−3(4)	−14(5)
C(27)	69(5)	39(3)	43(4)	−13(3)	−6(3)	−23(3)
C(28)	37(3)	30(3)	25(3)	−3(2)	0(2)	5(2)
C(29)	53(4)	26(3)	36(3)	−4(2)	5(3)	2(3)
C(30)	57(4)	37(3)	42(3)	0(3)	14(3)	−2(3)
C(31)	69(5)	38(3)	42(4)	3(3)	11(3)	−9(3)
C(32)	52(4)	29(3)	32(3)	1(2)	1(3)	10(3)
N(1)	26(2)	18(2)	24(2)	−2(2)	5(2)	−2(2)
N(2)	29(2)	26(2)	31(2)	−4(2)	6(2)	2(2)
N(3)	76(4)	27(3)	52(3)	1(2)	10(3)	0(3)
O(1)	28(2)	39(2)	41(2)	−6(2)	−2(2)	4(2)
O(2)	48(3)	48(2)	47(3)	−1(2)	−12(2)	18(2)
O(3)	49(3)	53(3)	34(2)	3(2)	−1(2)	−23(2)
O(4)	32(2)	41(2)	34(2)	0(2)	3(2)	−8(2)
O(5)	46(2)	26(2)	32(2)	−2(2)	5(2)	−3(2)
S(1)	33(1)	23(1)	24(1)	−2(1)	3(1)	−4(1)
